# Anti-inflammatory features of the amino acid-based formula Neocate

**DOI:** 10.1186/2045-7022-5-S3-P151

**Published:** 2015-03-30

**Authors:** Anita Hartog, Jacqueline Bastiaans, Nicole Buurman, Lucien Harthoorn, Hannah E Jones, Mona Bajaj-Elliott, A Paul Vos

**Affiliations:** 1 Nutricia Research, Utrecht, The Netherlands; 2 Nutricia Advanced Medical Nutrition, Utrecht, The Netherlands; 3 Institute of Child Health, London, United Kingdom

## 

Amino acid-based formulas (AAF) are used for the dietary management of cow’s milk allergy and multiple food protein intolerances. A recent study showed that intake of AAF, in addition to a milk-, egg-, wheat- and soy-free diet, results in a reduced colonic inflammatory status in pediatric patients (≤ 5 years, both control and formula group were negative for eosinophilia, [1] ). The present study aimed to evaluate the immune modulating effect of an amino acid-based formula (Neocate) and its pure total amino acid fraction.

Human peripheral blood mononuclear cells (PBMCs) were stimulated for 20 hours with lipopolysaccharide (LPS) in the presence and absence of the complete formula, its amino acid fraction, or individual amino acids. Cytokine production was analyzed in the culture supernatant, by ELISA. The influence on CXCL8-induced neutrophil chemotaxis was measured using Boyden chambers. AAF and its amino acid fraction significantly inhibit the LPS-induced TNFα production in a concentration dependent manner. However, the LPS-induced IL-8 production was not affected. In addition to the tested complete formula and amino acid fraction, the single amino acid glycine is able to inhibit the LPS-induced TNFα production. Other amino acids tested, including taurine, lysine and glutamine did not affect the LPS induced TNFα production. CXCL8-induced neutrophil chemotaxis was inhibited by the amino acids fraction and glycine but not by the complete amino acid-based formula.

The present study demonstrates that the amino acids in Neocate are able to dampen specific inflammatory responses. This effect might be mediated through specific amino acids or combinations thereof, including glycine. These *in vitro* studies show that the anti-inflammatory capacity of amino acid-based formulas (as indicated in [1] ) could, at least partially, be induced by the amino acid composition of the formula used.

## References

[B1] Jones HE Amino Acid-based Formula affects the gastrointestinal cytokine milieu of children with non-IgE mediated Cow’s Milk Allergy FAAM 2014

